# Detection of asymptomatic recurrence improves survival of gastric cancer patients

**DOI:** 10.1002/cam4.3899

**Published:** 2021-05-01

**Authors:** Ji Soo Park, Eun‐Ah Choe, Sejung Park, Chung Mo Nam, Woo Jin Hyung, Sung Hoon Noh, Seoyoung Lee, Hyo Song Kim, Minkyu Jung, Hyun Cheol Chung, Sun Young Rha

**Affiliations:** ^1^ Cancer Prevention Center Yonsei Cancer Center Yonsei University College of Medicine Seoul Republic of Korea; ^2^ Division of Medical Oncology Department of Internal Medicine Yonsei Cancer Center Yonsei University College of Medicine Seoul Republic of Korea; ^3^ Division of Hospital Medicine Department of Internal Medicine Yonsei University College of Medicine Seoul Republic of Korea; ^4^ Songdang Institute for Cancer Research Yonsei University College of Medicine Seoul Republic of Korea; ^5^ Department of Biostatistics and Computing Yonsei University College of Medicine Seoul Republic of Korea; ^6^ Department of Preventive Medicine Yonsei University College of Medicine Seoul Republic of Korea; ^7^ Department of Surgery Yonsei University College of Medicine Seoul Republic of Korea; ^8^ Brain Korea 21 Project for Medical Sciences Yonsei University College of Medicine Seoul Republic of Korea

**Keywords:** early detection of cancer, recurrence, stomach neoplasms, survivorship

## Abstract

**Background:**

The effect of long‐term surveillance for asymptomatic patients after curative resection of gastric cancer is being debated. We compared the prognosis of Korean patients with recurrent gastric cancer according to the presence or absence of cancer‐related symptoms at the time of recurrence detection.

**Methods:**

We retrospectively reviewed the medical records of 305 Korean patients who experienced recurrence after curative resection of primary gastric cancer between March 2002 and February 2017 at Yonsei Cancer Center, Yonsei University College of Medicine, Seoul, Republic of Korea.

**Results:**

The median follow‐up duration was 169.8 months (1–267.2), and the median age at first recurrence was 58.1 years (23.4–81.9). Among 305 patients with recurrence, 97 of 231 (42.0%) patients with early recurrence (≤5 years after curative surgical resection) and 47 of 74 (63.5%) patients with late recurrence (>5 years after curative surgical resection) had cancer‐related symptoms at recurrence (*p* = 0.001). For survival after recurrence, detection of asymptomatic recurrence was an independent favorable factor (hazard ratio, 0.527; 95% confidence interval, 0.409–0.681; *p* < 0.001) accompanied with the possibility of subsequent treatment, targeted‐, or immunotherapy for recurrent disease, and locoregional recurrence only. In the late‐recurrence group, the patients with asymptomatic detection of recurrence showed favorable post‐recurrence survival (median, 33.3 months vs. 14.7 months; *p* = 0.002), overall survival (median, 136.3 months vs. 106.1 months; *p* = 0.010), and cancer‐specific survival (median, 177.5 months vs. 106.1 months; *p* = 0.005) than the patients with symptomatic detection.

**Conclusion:**

The detection of gastric cancer recurrence in patients without cancer‐related symptoms may be related to improved survival, suggesting the potential benefit of long‐term surveillance.

## Graphical abstract

Asymptomatic detection of recurrence possibly improved the survival of gastric cancer long‐term survivors after curative surgical resection. The possibility of local control and targeted‐ or immunotherapy for the recurrent disease was related to the favorable survival outcomes.

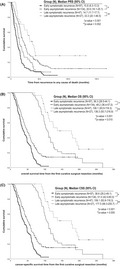

## 1 INTRODUCTION

Despite the incidence and mortality rates of gastric cancer decreasing in recent decades, it is still one of the most common cancers, particularly in East Asia.^1^ Meanwhile, with cancer screening and treatment advancements, the number of gastric cancer survivors also continues to increase. In Korea, gastric cancer patients have a 76.5% 5‐year survival rate, and long‐term (beyond 5 years from diagnosis) gastric cancer survivors exceeded 172,000 in 2017.^2^ Among the long‐term survivors, some patients experience cancer recurrence even 5–10 years after curative resection. However, although global clinical guidelines recommend active surveillance programs, including physical examination, endoscopy, blood tests, computed tomography, or ultrasonography within 5 years of the curative treatment of disease, most do not provide detailed surveillance recommendations after 5 years for long‐term survivors.^3^, ^4^, ^5^, ^6^ As late recurrence is uncommon in most cancer types, and there is insufficient evidence for active surveillance in the long‐term survivorship, surveillance and screening methods usually depend on physicians’ discretion in clinical practice.

In recent years, many researchers have demonstrated the lack of survival benefit of active surveillance for stomach cancer recurrence after curative resection.^7^, ^8^, ^9^, ^10^ Early detection of recurrence in asymptomatic patients facilitated prompt cancer treatment, resulting in a better clinical status; therefore, “asymptomatic patients” lived slightly longer than patients with late symptomatic detection. In contrast, symptomatic patients usually had a higher tumor burden than asymptomatic patients, as tumors grew and invaded organs during the symptom‐free period. Despite this, because the post‐recurrent survival benefit could not surpass the symptom‐free period of symptomatic patients, previous studies have not shown the overall survival (OS) benefit of asymptomatic detection of recurrence. However, several improvements have been made in the area of post‐recurrent treatment: chemotherapy, targeted therapy, immunotherapy, as well as endoscopic and surgical resection for gastric cancer. Due to the rapid increase in the number of gastric cancer survivors, there is a need to reconsider whether early detection of recurrence by active surveillance and subsequent cancer treatment might improve the survival of patients who underwent curative resection of primary gastric cancer.

In this study, we analyzed whether the survival of patients with recurrent gastric cancer was different according to the presence or absence of cancer‐related symptoms at the time of detection of recurrence. Especially, the correlation between symptomatic recurrence and survival outcomes was explored in both early (≤5 years after curative surgical resection) and late (>5 years after curative surgical resection) recurrence groups.

## 2 PATIENTS AND METHODS

### 2.1 Study population

This study included patients who had undergone curative resection of gastric cancer with recurrence between March 2002 and February 2017 at Yonsei Cancer Center, Yonsei University College of Medicine, Seoul, Republic of Korea. The criteria for eligibility were as follows: (i) histologically proven gastric or gastroesophageal adenocarcinoma, (ii) age of ≥18 years at the time of the first diagnosis of gastric cancer, (iii) operable disease at initial diagnosis and a history of curative surgical resection, and (iv) presence of recurrence confirmed by the pathological diagnosis of tumoral tissue in the recurrent site or definite image study.

Based on a prospectively compiled database, we enrolled 305 Korean patients who met the eligibility criteria and retrospectively reviewed their medical records. Information on sex, patient's age at diagnosis, histological classification, microsatellite instability status, human epidermal growth factor receptor 2 (HER2) status, TNM staging according to the American Joint Committee on Cancer staging manual 8th edition,^11^ time of recurrence, sites of recurrence, treatment for recurrence, survival duration, and cause of death were obtained. The protocol was approved by the institutional review board (IRB) of the Severance Hospital, Yonsei University Health System, Seoul, Republic of Korea (4‐2019‐0791). Informed consent was waived by the decision of IRB.

### 2.2 Definition of terms

Early recurrence was defined as recurrence detected within 5 years from the first curative resection of primary gastric cancer, whereas late recurrence was defined as recurrence detected beyond 5 years from the first curative resection.

Cancer‐related symptoms included aggravation of symptoms relevant to the recurrent site (e.g., cough or dyspnea for lung or pleural metastasis; nausea, vomiting, or severe abdominal distension for intestinal or peritoneal metastasis) as well as general symptoms of suspicious recurrence (e.g., abnormal weight loss and persistent, deteriorated, and poorly controlled pain). In this study, symptomatic recurrence was defined as the presence of cancer‐related symptoms reported by the patients when recurrence was detected.

Relapse‐free survival (RFS) was defined as the date of curative tumor resection to the first recurrence date. OS was defined as the date of first curative resection to the date of any cause of death. Cancer‐specific survival (CSS) was defined as the date of first curative resection to the date of death by gastric cancer progression. For the CSS analysis, death from other causes (e.g., acute infection, other primary malignancy, or suicide) was not considered an event. Post‐recurrence survival (PRS) was defined from the recurrence detection date to the date of any cause of death.

### 2.3 Adjuvant treatment and routine surveillance program

In this study, patients with high‐risk stage II or stage III disease were recommended to be treated with adjuvant chemotherapy. Adjuvant chemotherapeutic regimens included 5‐fluorouracil (5‐FU) or TS‐1 monotherapy, 5‐FU or TS‐1 plus cisplatin, capecitabine plus oxaliplatin, TS‐1 plus docetaxel, and 5‐FU plus doxorubicin combination therapy. After completion of curative surgical resection and/or adjuvant treatment, patients visited the clinic. They underwent routine follow‐up evaluation following the institutional protocol, which consisted of history taking, physical examination, hematological and chemistry tests, tumor marker detection, anemia profiling, chest radiography, abdominopelvic computed tomography, or sonography, every 3 months for the first 2 years and every 6 months thereafter for 5 years. Upper endoscopic evaluation (esophagogastroduodenoscopy) was performed annually. Beyond 5 years after curative resection, the gastric cancer survivors were recommended to visit the clinic annually for history taking, physical examination, blood tests, and endoscopic assessment. Imaging studies and further evaluation were considered based on cancer‐related symptoms, risk factors, and comorbidities of the patients.

### 2.4 Statistical analysis

This study's primary objective was to compare the patient OS according to the presence or absence of cancer‐related symptoms at the time of diagnosis of recurrence. The secondary objective was to compare the CSS and PRS in terms of cancer‐related symptoms at recurrence and the time of recurrence between the early‐ and late‐recurrence groups. Clinicopathological characteristics and treatment‐related factors were analyzed using Chi‐square, Fisher's exact, and Mann–Whitney *U* tests between the early‐ and late‐recurrence groups. The Kaplan–Meier method and Cox's proportional hazard model were used for survival analysis, and survival curves were compared using the log‐rank test. For survival analyses, stage of primary gastric carcinoma, type and time of recurrence, and subsequent treatment for the recurrent disease were also analyzed as the possible confounding variables. A *p* ˂ 0.05 was considered statistically significant. All statistical analyses were performed using SPSS 25 for Windows (IBM Corp.).

## 3 RESULTS

### 3.1 Differences in clinicopathological factors between the patients with early and late recurrence

Among 305 patients with recurrent gastric cancer, 231 patients (75.7%) were classified into the early‐recurrence group (within 5 years), and 74 patients (24.3%) into the late‐recurrence group (beyond 5 years). The median RFS duration of all patients, the early‐recurrence group, and the late‐recurrence group were 32.1 months (95% CI, 27.1–37.1), 22.5 months (95% CI, 20.2–24.8), and 87.9 months (95% CI, 80.0–95.4), respectively. Clinicopathological characteristics are shown in Table 1.

**TABLE 1:** Clinicopathological characteristics of the patients in early‐ and late‐recurrence group

Variables	Early‐recurrence (*N* = 231)		Late‐recurrence (*N* = 74)		*p*‐value
	*N*	%	*N*	%	
Sex					
Male	155	67.1%	52	70.3%	0.611
Female	76	32.9%	22	29.7%	
Characteristics of primary gastric carcinoma					
Age at diagnosis [years] (median, range)	55 (23.4–81.9)		51.2 (28.5–75.4)		0.269
TNM stage according to AJCC8					
I	27	11.7%	23	31.1%	<0.001^a^
II	33	14.3%	10	13.5%	
III	169	73.1%	30	40.5%	
Unknown	2	0.9%	11	14.9%	
Pathologic differentiation					
Adenocarcinoma, well differentiated	6	2.6%	8	10.8%	0.085
Adenocarcinoma, moderately differentiated	60	26.0%	19	25.7%	
Adenocarcinoma, poorly differentiated	108	46.8%	29	39.2%	
Signet ring cell carcinoma	41	17.7%	14	18.9%	
Others	16	6.9%	4	5.4%	
Lauren classification (*N* =265)					
Intestinal	85	38.5%	16	36.4%	0.840^a^
Diffuse	126	57.0%	27	61.4%	
Mixed	10	4.5%	1	2.2%	
MSI status (*N* = 164)					
MSI‐high	2	1.3%	1	7.7%	0.262^a^
MSI‐low	5	3.3%	0	0.0%	
MSS	144	95.4%	12	92.3%	
HER2 status (*N* = 258)					
Positive	15	7.1%	2	4.2%	0.747^a^
Negative	195	92.9%	46	95.8%	
Adjuvant chemotherapy					
Yes	163	70.6%	35	47.3%	0.001
No	68	29.4%	39	52.7%	
Characteristics of recurrence					
Age at recurrence [years] (median, range)	57.6 (26–82.5)		62.7 (33.7–83.3)		0.117
Site of recurrence					
Locoregional recurrence	19	8.2%	14	18.9%	0.010
Distant recurrence (multiple selection)					
Distant lymph nodes	47	20.3%	9	12.2%	0.114
Peritoneum	120	51.9%	28	37.8%	0.035
Krukenberg tumor	20	8.7%	4	5.4%	0.366
Liver	40	17.3%	11	14.9%	0.623
Lung	13	5.6%	10	13.5%	0.025
Bone	10	4.3%	11	14.9%	0.002
Pancreas	0	0%	4	5.4%	0.003^a^
Brain	0	0%	4	5.4%	0.003^a^
Others	2	0.9%	7	9.5%	0.001^a^
Presence of anemia^b^	126	54.5%	41	55.4%	0.897
Symptom at detection of recurrence					
Symptomatic	97	42.0%	47	63.5%	0.001
Asymptomatic	134	58.0%	27	36.5%	

Abbreviations: AJCC, American Joint Committee on Cancer; AMD, adenocarcinoma, moderately differentiated; APD, adenocarcinoma, poorly differentiated; AWD, adenocarcinoma, well differentiated; HER2, human epidermal growth factor receptor 2; MSI, microsatellite instability; MSS, microsatellite stable; SRC, signet ring cell carcinoma.

^a^ These values were analyzed by Fisher's exact test.

^b^ Anemia was defined as the hemoglobin level below 13 g/dl for males, below 12 g/dl for females.

The initial TNM stage of primary gastric cancer was slightly higher in the early‐recurrence group (stage I, II, and III: 11.7%, 14.3%, and 73.1%, respectively) than in the late‐recurrence group (*p* < 0.001). Although the proportion of the patients who were treated with adjuvant chemotherapy was higher in the early‐recurrence group (70.6%) than in the late‐recurrence group (47.3%, *p* = 0.001), the proportions only among the patients with stage II or III disease were not different between the two groups (81.1% in early‐recurrence group vs. 75.9% in late‐recurrence group, *p* = 0.515). Locoregional recurrence (limited to the stomach and regional lymph nodes) was more frequently found in the late‐recurrence group (18.9%) than in the early‐recurrence group (8.2%, *p* = 0.010). Peritoneal recurrence tended to be more frequent in the early‐recurrence group (51.9%) than in the late‐recurrence group (37.8%, *p* = 0.035). Among minor types of recurrence, the late‐recurrence group showed more frequent pancreatic metastasis (5.4% vs. 0%; *p* = 0.003) and brain metastasis (5.4% vs. 0%; *p* = 0.003) than the early‐recurrence group.

Among the late‐recurrence group, 17 patients experienced recurrence more than 10 years after the curative surgical resection of primary gastric cancer. The clinicopathological features are listed in Table S1. Most of the characteristics of the extremely late recurrence group (recurrence after >10 years) were not significantly different from those of the patients who experienced recurrence between 5 and 10 years. Anemia at detection of recurrence was more frequent in patients with recurrence after >10 years (82.4%) than in those with recurrence between 5 and 10 years (47.4%; *p* = 0.011).

Among 305 patients, 97 of 231 (42.0%) patients with early recurrence and 47 of 74 (63.5%) patients with late recurrence had cancer‐related symptoms at detection of recurrence (*p* < 0.001).

### 3.2 Survival outcomes according to the presence and absence of cancer‐related symptoms

**FIGURE 1:**
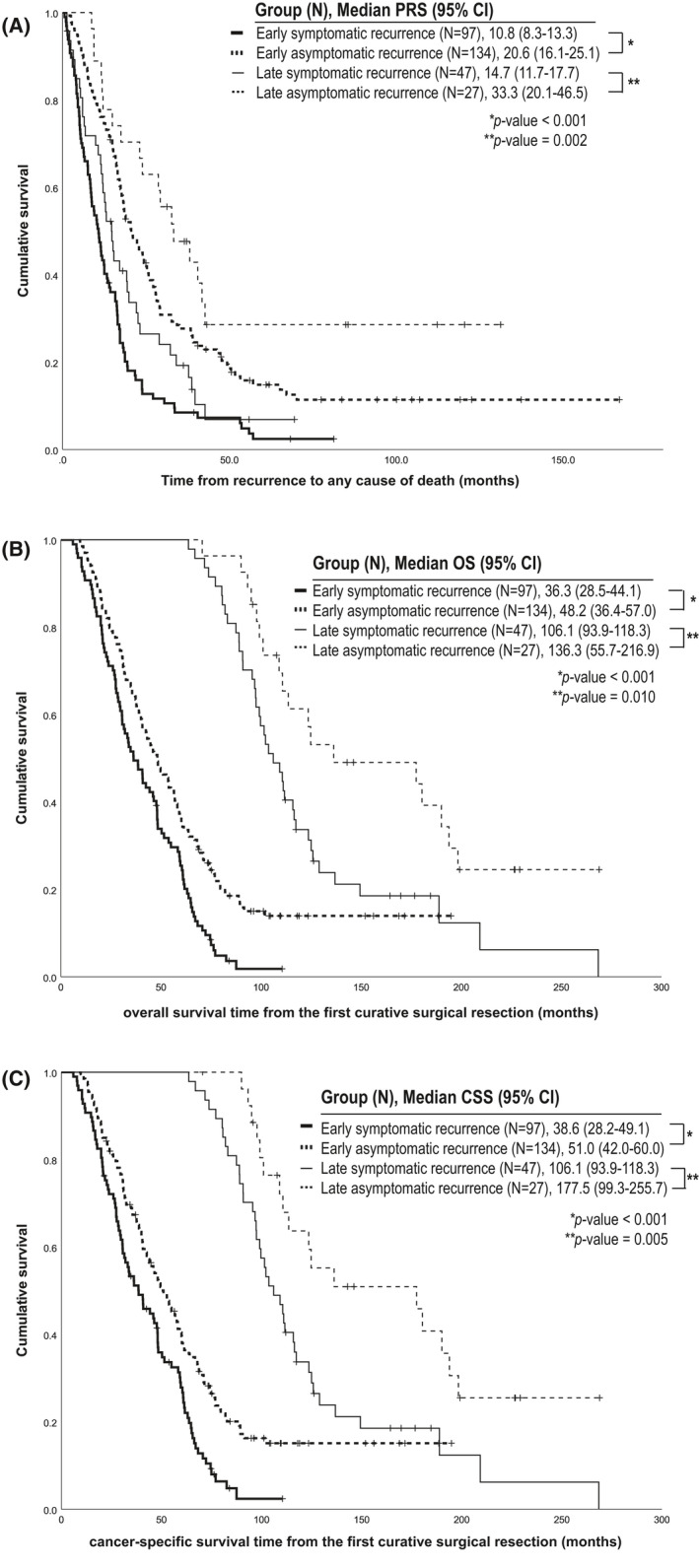
Survival outcomes in the early‐ and late‐recurrence groups according to the presence or absence of symptoms at the time of detection of gastric cancer recurrence. (A) The early‐recurrence group and late‐recurrence group were analyzed for post‐recurrence survival. (B) Both groups were analyzed for overall survival. (C) Both groups were analyzed for cancer‐specific survival. CSS, cancer‐specific survival; *N*, number; OS, overall survival; PRS, post‐recurrence survival

As of 31 December 2019, 206 patients in the early‐recurrence group (89.2%) and 58 patients in the late‐recurrence group (78.4%) died. The median follow‐up duration was 169.8 months (range, 6.0–267.2). Patients without symptoms at detection of recurrence lived longer than patients with symptoms at detection of recurrence in both subgroups of early recurrence (median PRS, 20.6 months [95% CI, 16.1–25.1] in asymptomatic vs. 10.8 months [95% CI, 8.3–13.3] in symptomatic patients; *p* < 0.001) and late recurrence (median PRS, 33.3 months [95% CI, 20.1–46.5] in asymptomatic vs. 14.7 months [95% CI, 11.7–17.7] in symptomatic patients; *p* = 0.002) (Figure 1A).

The OS was also longer in asymptomatic patients in the early‐recurrence group (median OS, 48.2 [95% CI, 36.4–57.0] in asymptomatic vs. 36.3 months [95% CI, 28.5–44.1] in symptomatic patients; *p* < 0.001) and in the late‐recurrence group (median OS, 136.3 [95% CI, 55.7–216.9] in asymptomatic vs. 106.1 months [95% CI, 93.9–118.3] in symptomatic patients; *p* = 0.010) (Figure 1B). In addition, the CSS was superior among the asymptomatic patients in the early‐recurrence group (median CSS, 51.0 months in asymptomatic patients vs. 38.6 months in symptomatic patients; *p* < 0.001) and in the late‐recurrence group (median CSS, 177.5 months in asymptomatic patients vs. 106.1 months in symptomatic patients; *p* = 0.005) (Figure 1C).

### 3.3 Prognostic value of the detection of asymptomatic recurrence considering subsequent treatment

Among a total of 305 patients, 144 (47.2%) patients had cancer‐related symptoms at recurrence (symptomatic detection group), and 161 (52.8%) patients did not have cancer‐related symptoms at recurrence (asymptomatic detection group). Differences in the clinical factors and treatment according to the presence of symptoms are listed in Table 2. There was a larger number of female patients (38.9% vs. 26.1%; *p* = 0.017), more peritoneal recurrence (61.1% vs. 37.3%; *p* < 0.001), more bone recurrence (11.8% vs. 2.5%; *p* = 0.001), and less locoregional recurrence (5.6% vs. 15.5%; *p* = 0.005) in the symptomatic detection group.

**TABLE 2:** Differences in clinical factors and treatment for recurrence according to the presence or absence of cancer‐related symptoms at the time of recurrence detection

Variables	Presence of symptom (*N* = 144)		Absence of symptom (*N* = 161)		*p*‐value
	*N*	%	*N*	%	
Sex					
Male	88	61.1%	119	73.9%	0.017
Female	56	38.9%	42	26.1%	
Age at recurrence [years] (median, range)	59.1 (26–83.3)		57.8 (33.5–82.5)		0.931
Site of recurrence					
Locoregional recurrence	8	5.6%	25	15.5%	0.005
Distant recurrence (multiple selection)					
Distant lymph nodes	27	18.8%	29	18.0%	0.868
Peritoneum	88	61.1%	60	37.3%	<0.001
Krukenberg tumor	12	8.3%	12	7.5%	0.776
Liver	19	13.2%	32	19.9%	0.119
Lung	13	9.0%	10	6.2%	0.352
Bone	17	11.8%	4	2.5%	0.001
Others	7	4.9%	2	1.2%	0.090^a^
Treatment for recurrent disease					
Operation only	0	0%	11	6.8%	0.002^a^
Chemotherapy only	94	65.3%	97	60.3%	
Radiotherapy only	4	2.8%	2	1.2%	
Multimodality	33	22.9%	45	28.0%	
CTx + OP (% of multimodality)	17	(51.5%)	31	(68.9%)	
RTx + OP (% of multimodality)	3	(9.1%)	0	(0%)	
Chemoradiation (% of multimodality)	12	(36.4%)	13	(28.9%)	
Chemoradiation + OP (% of multimodality)	1	(3.0%)	1	(2.2%)	
No (impossible or refuse)	13	9.0%	6	3.7%	
Surgical resection of recurrent tumor					
Operation with the aim of curative resection	6	4.2%	33	20.5%	<0.001
Palliative surgical resection	15	10.4%	10	6.2%	
Not done (impossible)	123	85.4%	118	73.3%	
Chemotherapy for recurrent cancer					
Done	124	86.1%	142	88.2%	0.586
Not done	20	13.9%	19	11.8%	
Line of chemotherapy	2 (1–5)		2 (1–8)		0.113
Use of targeted therapy or immunotherapy^b^					
Done	17	11.8%	25	15.5%	0.346
Not done	127	88.2%	136	84.5%	

Abbreviations: CTx, chemotherapy; OP, operation; RTx, radiotherapy.

^a^ This value was analyzed by Fisher's exact test.

^b^ These therapeutic drugs include trastuzumab, lapatinib, trastuzumab deruxtecan, ramucirumab, bevacizumab, onartuzumab, atezolizumab, pembrolizumab, nivolumab, afatinib, ipatasertib, olaparib, and other drugs currently under investigation.

In terms of treatment, 286 (93.8%) patients underwent treatment for recurrent gastric cancer. The asymptomatic detection group was more likely to be treated with curative or palliative surgical resection of the recurrent tumor (26.7% vs. 14.6% in symptomatic detection; *p* = 0.009). Local control, which was defined as treatment including surgical resection and/or radiotherapy of the recurrent tumor, was provided to 58 (36.0%) patients in the asymptomatic detection group and 37 (25.7%) patients in the symptomatic detection group (*p* = 0.042). Both groups received similar lines of chemotherapy (median, two cycles: *p *= 0.113). Forty‐two patients were also treated with targeted therapeutic or immunotherapeutic drugs, including drugs currently under investigation.

**FIGURE 2:**
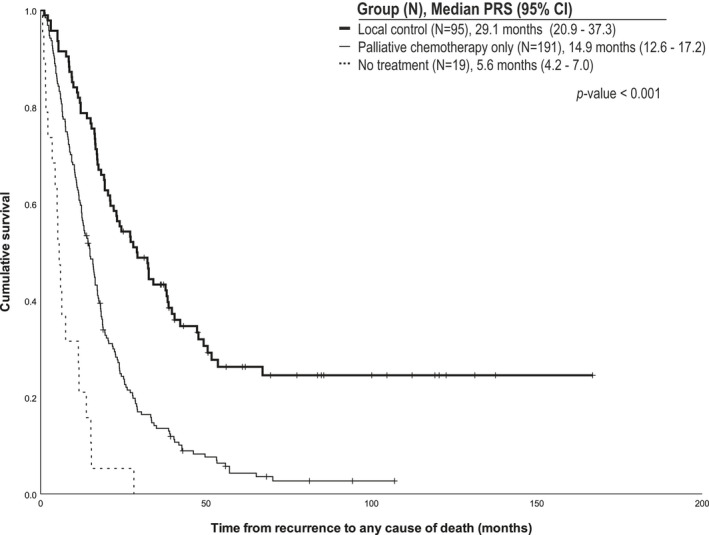
Post‐recurrence survival according to the type of treatment for recurrent disease. PRS, post‐recurrence survival

After adjustment of several confounding variables, the detection of asymptomatic recurrence was found to be a favorable prognostic factor for PRS (HR, 0.527; 95% CI, 0.409–0.681; *p* < 0.001). The detection of recurrence in patients without cancer‐related symptoms was also related to improved CSS (HR, 0.675; 95% CI, 0.513–0.886; *p* = 0.005) and OS (HR, 0.689; 95% CI, 0.528–0.899; *p* = 0.006; Table 3). In terms of the treatment method for recurrent disease, local control (HR, 0.154; 95% CI, 0.090–0.265; *p* < 0.001) and targeted‐ or immunotherapy (HR, 0.498; 95% CI, 0.341–0.727; *p* < 0.001) were related to favorable PRS, conferring superior CCS and OS (Table 3). The median number of PRS patients who underwent local control treatment (*N* = 95), underwent palliative chemotherapy only (*N* = 191), and without treatment for recurrent disease (*N* = 19) were 29.1 months (95% CI, 20.9–37.3), 14.9 months (95% CI, 12.6–17.2), and 5.6 months (95% CI, 4.2–7.0), respectively (*p* < 0.001; Figure 2).

**TABLE 3:** Clinical factors and treatment for recurrence with significance in terms of survival outcomes (using Cox hazard multiple regression model) (*N* = 305)

Variables	*N*	Post‐recurrence survival			Cancer‐specific survival			Overall survival		
		HR	95% CI	*p*‐value	HR	95% CI	*p*‐value	HR	95% CI	*p*‐value
Characteristics of primary gastric carcinoma										
Stage of primary gastric cancer (AJCC 8th edition) (*N* = 292)										
I or II	93				1			1		
III	199				1.411	1.060–1.880	0.018	1.482	1.118–1.965	0.006
Characteristics of recurrence										
Type of recurrence										
Locoregional recurrence only	33	1			1			1		
Distant or multiple recurrence	272	1.961	1.171–3.284	0.010	1.748	1.022–2.990	0.041	1.758	1.044–2.960	0.034
Time of recurrence										
Early recurrence (≤5 years after curative surgical resection)	231				1			1		
Late recurrence (>5 years after curative surgical resection)	74				0.200	0.136–0.294	<0.001	0.201	0.138–0.294	<0.001
Presence of cancer‐related symptom at the time of diagnosis of recurrence										
Presence (symptomatic)	144	1			1			1		
Absence (asymptomatic)	161	0.527	0.409–0.681	<0.001	0.675	0.513–0.886	0.005	0.689	0.528–0.899	0.006
Subsequent treatment for recurrent disease										
Type of treatment for recurrent disease										
Not done (impossible or refuse)	19	1			1			1		
Chemotherapy only (palliative)	191	0.271	0.164–0.449	<0.001	0.641	0.394–1.042	0.073	0.676	0.417–1.097	0.113
Local control for recurrent tumor^a^	95	0.154	0.090–0.265	<0.001	0.303	0.176–0.519	<0.001	0.334	0.196–0.570	<0.001
Targeted‐ or immunotherapy for recurrent gastric cancer										
Not done	263	1			1			1		
Done	42	0.498	0.341–0.727	<0.001	0.626	0.425–0.922	0.018	0.617	0.422–0.903	0.013

Abbreviation: HR, hazard ratio.

^a^ Local control was defined as the treatment including surgical resection and/or radiotherapy of the recurrent tumor.

In addition, we observed a few long‐term survivors after treatment for recurrent disease. Twelve of 161 patients with asymptomatic recurrence (7.5%) and one of 144 patients with symptomatic recurrence (0.7%) are still alive without any clinical evidence of residual tumors (*p* = 0.004) for a median of 100.1 months (range, 36.5–166.9) since the first recurrence of gastric cancer. Among the 13 long‐term survivors, seven patients underwent completion of total gastrectomy for the lesions limited in the stomach, three patients underwent metastasectomy (liver and ovary) followed by chemotherapy, two patients were treated with concurrent chemoradiation therapy, and one patient experienced the disappearance of a metastatic nodule during continuous chemotherapy.

## 4 DISCUSSION

In the present study, we demonstrated that the detection of gastric cancer recurrence after curative surgical resection in patients without cancer‐related symptoms might be related to favorable OS, CSS, and survival after recurrence in both the early‐ and late‐recurrence groups.

Early detection of primary cancer usually increases the efficacy of treatment. A possible explanation is that localized disease can be treated with curative surgical or endoscopic resection. Furthermore, even in unresectable disease, the performance status and major organ function are suitable for active systemic treatment. Similarly, active surveillance is recommended for the early detection of cancer recurrence after curative surgical resection. However, the possibility of surgical resection of metastatic tumors and the efficacy of subsequent chemotherapy were limited in patients with gastric cancer until recently. Moreover, most recurrent cases happen within 5 years after curative surgical resection; both physicians and patients pay less attention to gastric cancer recurrence beyond 5 years after curative surgical resection. Therefore, active surveillance for asymptomatic long‐term gastric cancer survivors is uncommon in clinical practice. In this study, the detection of recurrence without cancer‐related symptoms was more common in the early‐recurrence group (58.0%) than in the late‐recurrence group (36.5%, *p* = 0.001), which is probably due to the greater number of active surveillance programs recommended,^3^, ^4^, ^5^, ^6^, ^10^ and applied within 5 years after curative surgical resection. The changing proportion of asymptomatic detection over time possibly affected the survival analysis of the patients according to the symptom. Our data showed that the median OS duration was 59.7 months (95% CI, 47.8–71.6) in all the patients with symptomatic recurrence and 57.7 months (95% CI, 50.1–65.3) in all the patients without symptomatic recurrence (*p* = 0.111). However, the results of the time‐dependent Cox model (HR, 0.640; 95% CI, 0.495–0.827; *p* = 0.001) subgroup analysis in the early‐ and late‐recurrence group (Figure 1) and multiple regression analysis (Table 3) represented the significance of detection of asymptomatic recurrence. Therefore, although the OS outcome of late asymptomatic recurrence did not exceed those of early symptomatic recurrence (Figure 1B,C), we suggest that the effort for detection of late recurrence at the time of asymptomatic status could be considered in the group of long‐term gastric cancer survivors beyond 5 years from curative resection.

Several recent studies showed that the detection of asymptomatic recurrence was related to improved survival outcomes,^11^, ^12^ in contrast to other reports.^7^, ^8^, ^9^ Considering that even the studies not showing the efficacy of detecting asymptomatic recurrence generally agreed that there is a correlation between asymptomatic recurrence and prolonged survival after recurrence, the treatments for recurrent disease possibly affected the different survival outcomes. Therefore, in this study, we analyzed survival outcomes not only according to the presence of symptoms but also the subsequent treatment for recurrent disease, the stage of primary cancer, and characteristics of recurrence. As a result, the possibility of surgical resection of recurrent tumors was related to improved OS (HR, 0.454; 95% CI, 0.310–0.664, *p* = 0.002), CSS (HR, 0.432; 95% CI, 0.291–0.642, *p* < 0.001), and prolonged PRS (HR, 0.460; 95% CI, 0.316–0.670, *p* < 0.001). Even local control of the recurrent tumor with curative or palliative treatment was related to prolonged OS (HR, 0.334; 95% CI, 0.196–0.570, *p* < 0.001), CSS (HR, 0.303; 95% CI, 0.176–0.519, *p* < 0.001), and PRS (HR, 0.154; 95% CI, 0.090–0.265, *p* < 0.001; Table 3). In addition, recent advances in novel treatment options, including the targeted therapies for HER2,^13^ vascular endothelial growth factor 2 (VEGFR2),^14^, ^15^ and immunotherapy^16^ for recurrent disease, possibly improved the survival outcomes. In addition, because the second and subsequent chemotherapy lines were also found to be effective and tolerable to patients,^17^, ^18^ active treatment for the recurrent disease was applied more frequently than before. In fact, in this study, the patients who were treated with targeted therapy, including HER2 monoclonal antibody, VEGFR2 monoclonal antibody, and immunotherapy, showed superior survival outcomes compared to those who were not treated with such methods (HR for OS, 0.617; 95% CI, 0.422–0.903; *p* = 0.013; Table 3). These active treatments for recurrent disease probably improved PRS of the asymptomatic patients with locoregional or oligometastatic lesions. The improvement in PRS could result in prolonged CSS and OS.

Much more recent studies reported that late recurrence of gastric cancer occurred beyond 5 and even 10 years after curative surgical resection of primary tumor.^19^, ^20^, ^21^, ^22^ The present study also showed that late recurrence occurred in 74 patients. Among them, 17 (23.0%) patients were diagnosed with recurrent gastric cancer after >10 years since curative surgical resection (Table 1). Half of these extremely late recurrence cases (8/17, 47.1%) were asymptomatic and detected during the follow‐up screening. Even though the numbers are few (17/305, 5.6%), considering these extremely late recurred cases exist, we need to follow‐up with long‐term survivors. In addition, we suggested the relationship between the detection of recurrence in the patients without cancer‐related symptoms and improved survival outcomes of gastric cancer patients, even among the long‐term survivors. Even so, whether an active surveillance program similar to the one within 5 years after curative surgical resection is necessary for long‐term survivors is doubtful because late recurrence is still rare compared to early recurrence, and the loci or onset of late recurrence is hard to predict. Many researchers suggested prognostic factors for late recurrence, including initial stage IV,^21^ inversely lower stage and lesser lymph node invasion than early recurrence,^22^, ^23^ younger age,^20^, ^23^ and larger tumor size.^20^ In this study, ages at diagnosis of primary gastric carcinoma were not related to the recurrence‐free survival in both early‐ and late‐recurrence groups. Late recurrence was more frequent in patients with early‐stage primary gastric cancer and was more likely found as locoregional, lung, or bone recurrence (Table 1). Many researchers have suggested that cancer dormancy could be a strong candidate for the culprit of late recurrence.^24^ A previous study hypothesized “cancer without disease” in an unrecognized patient as local malignant dormancy,^25^ and the lung, bone marrow, and brain as the candidates for the tissue‐specific perivascular dormant niche of disseminated tumor cells.^26^ In this respect, site‐specific late recurrence was possibly related to cancer dormancy and its microenvironments. However, at present, the accurate prediction of late recurrence and personalized surveillance are still difficult to apply in clinical practice. Further investigations on more cases of late recurrence are needed.

This study has several limitations. First, we could not assess the quality‐adjusted life years. The importance of survival with a good quality of life becomes more highlighted for cancer survivors. If the patients with asymptomatic recurrence can live longer than the patients with cancer‐related symptoms at detection of recurrence, treatment for recurrent disease can relieve cancer‐related symptoms and maintain the patients’ quality of life. Second, a routine surveillance program for long‐term survival was not applied. Most asymptomatic recurrences were observed by elevated tumor markers or incidental findings in the imaging study to screen other cancers or diseases. Several symptomatic recurrences were observed in the patients who were lost to follow‐up. Because cancer‐related symptom was subjectively felt and expressed by the patients, an objective analysis was difficult. Therefore, an objective risk assessment and long‐term surveillance program should be developed and estimated. Adequate surveillance methods, intervals, and risk models in predicting recurrence could not yet be established through this study.

Despite these limitations, this study can provide a timely reminder regarding the surveillance for recurrence, especially in long‐term survivors of gastric cancer, in the era of long‐term survivorship. Based on the OS benefit of asymptomatic detection and clinical characteristics of late recurrence, we can conceive an active surveillance program beyond 5 years. A personalized prediction model for late recurrence, including molecular or genetic factors, and cost‐effective and sensitive screening methods are needed to be developed in future investigations.

## CONFLICT OF INTEREST

The authors declare that they have no conflict of interest.

## ETHICS STATEMENT

All procedures followed were in accordance with the ethical standards of the responsible committee on human experimentation (institutional and national) and with the Helsinki Declaration of 1964 and later versions.

## Supporting information

Table S1

## Data Availability

The data that support the findings of this study are available on request from the corresponding author. The data are not publicly available due to privacy or ethical restrictions.
