# Supplementary material for: Detection of asymptomatic recurrence improves survival of gastric cancer patients
Source: Cancer Med. 2021 May 1;10(10):3249–60. doi: 10.1002/cam4.3899 (PMC8124119; doi:10.1002/cam4.3899)
Supplement: Supplementary file 1 — Table S1 [file CAM4-10-3249-s001.docx]

**Supplementary Table 1.** Comparison of clinicopathologic characteristics between late and extremely late recurrence

| **Variables** | | **Recurrence >5 Y and≤10 Y after  curative resection**  **(N=57)** | | **Recurrence >10 Y after  curative resection**  **(N=17)** | |  |
| --- | --- | --- | --- | --- | --- | --- |
|  | | **N** | **%** | **N** | **%** | ***p*- value** |
| Sex | |  |  |  |  |  |
|  | Male | 37 | 64.9% | 15 | 88.2% | 0.065 |
|  | Female | 20 | 35.1% | 2 | 11.8% |  |
| *Characteristics of primary gastric carcinoma* | | | | | | |
| Age at diagnosis [years] (median, range) | | 50.3(28.5-75.4) | | 51.2(38.2-63.8) | | 0.969 |
| TNM stage according to AJCC8 | |  |  |  |  |  |
|  | I | 16 | 28.1% | 7 | 41.2% | 0.348^*^ |
|  | II | 9 | 15.8% | 1 | 5.9% |  |
|  | III | 25 | 43.8% | 5 | 29.4% |  |
|  | unknown | 7 | 12.3% | 4 | 23.5% |  |
| Pathologic differentiation | |  |  |  |  |  |
|  | Adenocarcinoma, well differentiated | 7 | 12.3% | 1 | 5.9% | 0.231^*^ |
|  | Adenocarcinoma, moderately differentiated | 12 | 21.1% | 7 | 41.2% |  |
|  | Adenocarcinoma, poorly differentiated | 21 | 36.8% | 8 | 47.1% |  |
|  | Signet ring cell carcinoma | 13 | 22.8% | 1 | 5.9% |  |
|  | Others | 4 | 7.0% | 0 | 0% |  |
| Lauren classification (N=44) | |  |  |  |  |  |
|  | Intestinal | 13 | 34.2% | 3 | 50.0% | 0.702^*^ |
|  | Diffuse | 24 | 63.2% | 3 | 50.0% |  |
|  | Mixed | 1 | 2.6% | 0 | 0% |  |
| MSI status (N=13) | |  |  |  |  |  |
|  | MSI-high | 0 | 0% | 1 | 100% | 0.077^*^ |
|  | MSI-low | 0 | 0% | 0 | 0% |  |
|  | MSS | 12 | 100% | 0 | 0% |  |
| HER2 status (N=48) | |  |  |  |  |  |
|  | positive | 1 | 2.7% | 1 | 9.1% | 0.410^*^ |
|  | negative | 36 | 97.3% | 10 | 90.9% |  |
| *Characteristics of recurrence* | |  | |  | |  |
| Age at recurrence [years] (median, range) | | 56.9(33.7-83.3) | | 65.3(48.6-79.0) | | 0.075 |
| Site of recurrence | |  |  |  |  |  |
|  | Locoregional recurrence | 10 | 17.5% | 4 | 23.5% | 0.725^*^ |
|  | Distant recurrence (multiple selection) |  |  |  |  |  |
|  | Distant lymph nodes | 8 | 14.0% | 1 | 5.9% | 0.675^*^ |
|  | Peritoneum | 23 | 40.4% | 5 | 29.4% | 0.414 |
|  | Krukenberg tumor | 4 | 7.0% | 0 | 0% | 0.568^*^ |
|  | Liver | 7 | 12.3% | 4 | 23.5% | 0.263^*^ |
|  | Lung | 8 | 14.0% | 2 | 11.8% | >0.99 |
|  | Bone | 11 | 19.3% | 0 | 0% | 0.059^*^ |
|  | Others | 6 | 10.5% | 1 | 5.9% | >0.99 |
| Presence of anemia^†^ | | 27 | 47.4% | 14 | 82.4% | 0.011 |
| Symptom at detection of recurrence | |  |  |  |  |  |
|  | Symptomatic | 38 | 66.7% | 9 | 52.9% | 0.302 |
|  | Asymptomatic | 19 | 33.3% | 8 | 47.1% |  |

*this value was analyzed by Fisher’s exact test

^†^Anemia was defined as the hemoglobin level below 13 g/dL for males, below 12 g/dL for females

Abbreviations, AJCC, American Joint Committee on Cancer; AMD, adenocarcinoma, moderately differentiated; APD, adenocarcinoma, poorly differentiated; AWD, adenocarcinoma, well differentiated; HER2, human epidermal growth factor receptor 2; MSI, microsatellite Instability; MSS, microsatellite stable; SRC, signet ring cell carcinoma; Y, years;
